# Evolution and diversification of the nuclear pore complex

**DOI:** 10.1042/BST20200570

**Published:** 2021-07-20

**Authors:** Alexandr A. Makarov, Norma E. Padilla-Mejia, Mark C. Field

**Affiliations:** 1 School of Life Sciences, University of Dundee, Dundee DD1 5EH, U.K.; 2 Institute of Parasitology, Biology Centre, Czech Academy of Sciences, 37005 České Budějovice, Czech Republic

**Keywords:** eukaryogenesis, evolutionary biology, nuclear pores, nuclear protein transport

## Abstract

The nuclear pore complex (NPC) is responsible for transport between the cytoplasm and nucleoplasm and one of the more intricate structures of eukaryotic cells. Typically composed of over 300 polypeptides, the NPC shares evolutionary origins with endo-membrane and intraflagellar transport system complexes. The modern NPC was fully established by the time of the last eukaryotic common ancestor and, hence, prior to eukaryote diversification. Despite the complexity, the NPC structure is surprisingly flexible with considerable variation between lineages. Here, we review diversification of the NPC in major taxa in view of recent advances in genomic and structural characterisation of plant, protist and nucleomorph NPCs and discuss the implications for NPC evolution. Furthermore, we highlight these changes in the context of mRNA export and consider how this process may have influenced NPC diversity. We reveal the NPC as a platform for continual evolution and adaptation.

## Introduction

**Figure 1.**
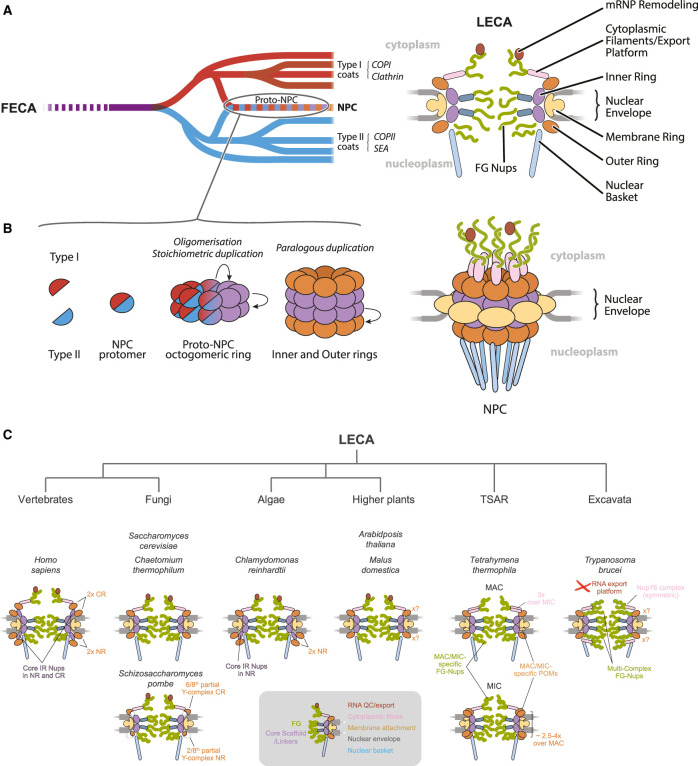
Evolutionary origins of the NPC. (**A**) Protocoatomer hypothesis states that a single protocoatomer (left, dark purple line) originated sometime early in eukaryotic evolution and gave rise to the two major coat protein families — type I (red lines) and type II (blue lines). Type I and type II coat proteins jointly formed the Proto-NPC — progenitor of the LECA NPC (right) — the concentric assembly of octagonally symmetric inner (IR, purple and dark blue), outer (OR, orange) and membrane (MR, beige) rings anchored in the NE pore that houses the nuclear basket (light blue), the cytoplasmic export platform (pink and burgundy) and FG-repeat nucleoporins (green). (**B**) An assembly of type I and II coat proteins formed an NPC protomer that populated the double inner ring via oligomerisation and stoichiometric duplication, and further — via paralogous duplication — the outer rings of LECA NPC. (**C**) Further diversification post-LECA gave rise to many NPC architectures in major taxa of eukaryotic tree (top) that principally differ in the stoichiometry and symmetry of outer cytoplasmic and nucleoplasmic rings (CR and NR), the manner of outer ring attachment to the inner rings and presence and symmetry of specific FG-nup and MR elements.

The nuclear pore complex (NPC), responsible for bidirectional transport of proteins and RNA between the cytosol and nucleus, is an octagonally symmetric structure consisting of multiple co-axial rings, each built of eight spokes (Figure 1). The inner (IR) and outer cytoplasmic and nuclear rings (CR and NR) form the core scaffold, anchored to membrane ring (MR) *trans*-membrane domain proteins and housing the bulk of FG-nucleoporins (Nups) that maintain the NPC permeability barrier. The nuclear basket (NB) and cytoplasmic filaments (CFs) are attached to the core scaffold and contribute towards both protein and mRNA transport [1].

The protocoatomer hypothesis [2] was proposed in recognition of common architectures between core proteins of multiple complexes within eukaryotic cells, including the NPC. Protocoatomers are membrane-deforming proteins consisting of β-propellers and α-solenoids, and while the primary structure between family members is frequently poorly retained, the secondary structure is considerably better conserved. Inferred from this is that an archetypal membrane coating complex evolved in the earliest eukaryotes (Figure 1A), supported by the presence of β-propeller and α-solenoid-encoding genes within the closest known prokaryotic ancestors of eukaryotes, the archaea. The model proposes that through paralogous duplication, extensive type I and II coat families arose: Type I coats feature an N-terminal β-propeller followed by a continuous α-solenoid, while type II coats bear a perversion within the α-solenoid [3,4]. NPCs contain proteins of both type I and II architectures, suggesting evolution followed the establishment of the major coat types. In *Saccharomyces cerevisiae* two clearly paralogous columns parallel to the NPC axis form each IR spoke and each spoke is, in turn, duplicated vertically. Similarly, the spokes in the CR and NR are built of two columns each. The column building block is an amalgam of type I and II coat proteins, and this subcomplex, via paralogous expansions, possibly populated the NPC architecture in the LECA (Figure 1B).

Molecular data are available for NPCs from many lineages, including metazoa (*Homo sapiens*) [5–8], fungi (*S. cerevisiae*, *Schizosaccharomyces pombe*, *Chaetomium thermophilum*) [9–16], algae (*Chlamydomonas reinhardtii*) [17], higher plants (*Arabidopsis thaliana, Malus domestica*) [18,19], alveolates (*Tetrahymena thermophila*) [20] and excavates (*Trypanosoma brucei*) [21]. While not particularly deep in taxon coverage, these data span a considerable proportion of eukaryotic diversity. Here we consider the structural variations that are evident between NPCs, consider export mechanisms for transiting the pore and how these systems co-evolved.

## Stoichiometry in outer rings

**Figure 2.**
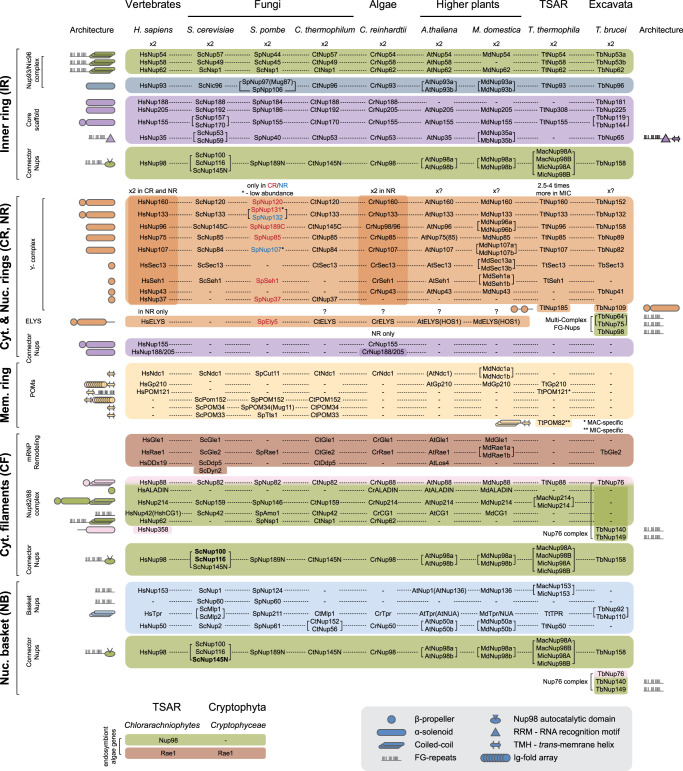
Comparisons of nucleoporins across species in selected taxa. Data collated for *H. Sapiens* [8], *S. cerevisiae* [10], *S. pombe* [12], *C. thermophilum* [14], *C. reinhardtii* [17], *A. thaliana* [18], *M. domestica* [19], *T. thermophila* [20], *T. brucei* [21] species and *Chlorarachniophytes* and *Cryptophyceae* species clades [125]. Nucleoporins are listed according to their complex and ring disposition in NPC compartments: the inner ring (IR), outer cytoplasmic and nucleoplasmic rings (CR and NR), membrane ring (Mem. Ring, MR), cytoplasmic filaments (Cyt. Filaments, CF) and nuclear basket (Nuc. Basket, NB). Nucleoporins are additionally coloured by type: FG-nups and linker FG-nups in IR, CR and NR, CF and NB in swamp green; Nup93/Nic96 complex core in IR in blue; core IR scaffold Nups in purple (also present in ORs as connector Nups); OR Nups in orange; pore membrane proteins (POMs) in MR in beige; Cytoplasmic Export platform scaffold in CF in pink; mRNP Remodelling in CF in burgundy, and basket scaffold Nups in NB in light blue. Each row represents an orthologous nucleoporin group. Nucleoporins absent in an organism are indicated by a dash (-). In cases of paralogous duplication within an organism — multiple nucleoporins are listed in square brackets, i.e. [ScNup167 ScNup170]. Alternative nucleoporin names are listed in round brackets, i.e. HsNup42 (HshCG1). Ring stoichiometry noted for IR and ORs. Exclusive Y-complex component distribution in *S. pombe* is additionally indicated by colour: red for CR-specific and blue for NR-specific. Macronuclei/micronuclei- (MAC-/MIC-) specificity for nucleoporins in *Tetrahymena* is denoted by Mac- or Mic-prefixes in gene names or * and ** for POMs. *Trypanosoma*-specific Multi-Complex FG-Nup and Nup76 complexes are shown by additional brackets. In ‘architecture’ column (left) given are the schematic protein fold architectures common for the orthologous Nup groups. Additional unique architectures are indicated for TbNup65 that sports a *trans*-membrane helix uniquely in its orthologous group (demonstrated on the right), and for lineage-specific Nups TtPOM82, TtNup185, TbNup64, TbNup75, TbNup98, TbNup140 and TbNup149.

The outer rings anchor the cytoplasmic export platform and nuclear basket [6,10,11,22–24]. The Nup85 (yeast nomenclature) or Y-complex is the outer ring building block and contains up to nine components. All are β-propeller, α-solenoid or β-propeller/α-solenoid proteins, archetypal for membrane coating complexes and thus likely to share a common evolutionary origin [1,25–27]. At least six Y-complex components are broadly conserved and five are scaffold nucleoporins, orthologs of HsNup75, HsNup96, HsNup107, HsNup133 and HsNup160 [28]. Notable exceptions are *T. brucei* and *T. thermophila*; each possess novel or highly divergent β/α-proteins TbNup109 [21] and TtNup185 (albeit with some similarity to HsNup133, 155 and 160), respectively [20]. The remaining components are β-propeller nucleoporins, the widely conserved Sec13, Seh1 (absent from excavates), HsNup43 (absent from fungi and TSAR) and HsNup37 (restricted to animals and some fungi); all of which suggests considerable evolutionary flexibility (Figure 2).

Notably, a pronounced difference in NPC organisation across taxa lies in the number of these rings and stoichiometry of Y-complex components. While *S. cerevisiae* has a single CR and NR [10], *H. sapiens* enjoys two copies of each [6] and curiously *C. reinhardtii* has two NR but only one CR [17] (Figures 1C and 2). Additional components are present for interconnection of these duplicate rings, including the vertebrate-specific CF component RanBP2/Nup358 [7,29] and inner ring components — Nup155, 188 and 205 [6–8,17,29]. Deviating further are *S. pombe* and *T. thermophila* (Figures 1C and 2). *S. pombe* rings are *‘*split’: SpNup107 and SpNup132 localise exclusively to the NR, while six remaining nucleoporins, including SpNup131 (diverse paralog of SpNup132), are CR-exclusive. Deepening this uneven distribution is the overall comparatively low abundance of SpNup107 and SpNup131 [12,13]. *T. thermophila* NPCs, by contrast, demonstrate an uneven distribution but with comparatively higher abundance of 2.5- to 4-fold for Y-complex subunits in the micronucleus (MIC) [20]. Whether this is related to the transcriptional activity differences is yet to be explored. *M. domestica* (apple) differs from *Arabidopsis* as it possesses multiple Y-complex subunit paralogs (albeit likely results of genome duplication) [18,19] (Figure 2), while *T. brucei* acquired an additional complex of three FG-Nups, symmetrically positioned on both sides of the NE [21]; the exact symmetry and stoichiometry of the outer ring in plants and excavates remains to be established.

ELYS, a β/α-nucleoporin with a nucleosome-binding domain, originally identified as a transcription factor [30], interacts with both the Y-complex and pore membrane proteins (POMs). ELYS mediates post-mitotic NPC assembly/anchoring, chromatin compaction and NPC/lamina associations, but most of these functions are metazoan and/or open mitosis-specific [31,32]. Paradoxically ELYS is present in most taxa, except excavates or TSAR, suggesting a probable ancient origin [12,17–21]. As neither plants nor fungi share a lamina with metazoa, ELYS functions are unclear [33,34]. Even more unusual is that the *S. pombe* ELYS ortholog is located on the cytoplasmic side of the NPC, potentially precluding obvious nucleoplasmic roles [13].

## Expansion, loss and diversification in the inner core

Similar to ORs, main IR components are large α-solenoid (Nup93/Nic96, Nup188, Nup205 orthologs) or β-propeller/α-solenoid proteins (Nup155 orthologs) and again with clear origins in membrane coating complexes [1,25–27]. The IR is well conserved but sports surprising functional diversity (Figure 2). IR in some taxa rely on nucleoporins containing a membrane-binding domain (MMB) for anchorage to the pore membrane. Of these ScNup53 and ScNup59 in *S. cerevisiae* and MdNup35a and MdNup35b in *M. domestica* exemplify a paralog duplication absent elsewhere and presumably occurred independently. However, *T*. *brucei* sports TbNup65, a single ortholog to ScNup53/59, possessing a conventional *trans*-membrane domain instead of an amphipathic lipid-packing sensor (ALPS) MMB present in ScNup53/59 [21]. *T. thermophila*, by contrast, has no identifiable orthologs to ScNup53/59 and possibly relies on TtNup155, an ortholog to ScNup157/170, and additional POMs for membrane anchoring [20].

Connections between the IR, OR, NB and CFs are also variant. In *S. cerevisiae* connector FG-nups ScNup145N/ScNup116/ScNup100 asymmetrically connect IR with outer rings, with ScNup145N extending from the central NPC towards NR and NB, and ScNup116 and ScNup100 binding IR to CR and the export complex [10] (Figure 2). Furthermore, ScNup157 and 170 stabilise the spokes within the inner ring, while ScNup188 and ScNup192 act as buttresses within the spokes and neither interact with the OR. However, to facilitate connections with duplicate ORs in *H*. *sapiens* and *C. reinhardtii* eight additional copies of HsNup155/CrNup155 and HsNup188/CrNup188 (respective orthologs of ScNup157/170 and ScNup188) form pillars, one per spoke, on each side of the NE when there are duplicate ORs [6–8,17]. Interestingly, in the *Xenopus laevis* NPC XlNup205 (ortholog of ScNup192) replaces Nup188 [29], which suggests considerable flexibility, even within vertebrates.

Unsurprisingly, excavate and TSAR NPCs are organised distinctly to metazoan and fungal complexes (Figure 2). *T. brucei* has a paralog pair, TbNup144 (orthologous to ScNup157/170) and TbNup119 (similar to ScNup170) [21]. Notably, pullouts revealed TbNup144 to have weak interactions with TbNup89, the ortholog of HsNup75/ScNup85 of the Y-complex. In contrast TbNup119 pulled down the entire *T. brucei* OR as well as the IR core α-solenoid TbNup225, the ortholog of HsNup205/ScNup192. Notably, neither TbNup225, nor other components of the T*. brucei* IR interact with the OR, suggesting that TbNup119 bridges IR and OR and is more akin to ScNup145N/ScNup116/ScNup100. Simultaneously no data exists on whether TbNup158, an ortholog to HsNup98/(ScNup145N/ScNup116/ScNup100), is present in the IR or contributes to IR-OR connection. Instead, TbNup158 appears as a constituent component of the Y-complex in *T. brucei* anchoring the TbNup76 complex and multi-complex FG-Nups in the ORs [21]. Finally, T*. thermophila* sports single orthologs to each HsNup155/(ScNup157/ScNup170)/(TbNup144/TbNup199) and HsNup205/ScNup192/TbNup225, but up to four orthologs to HsNup98/(ScNup145N/ScNup116/ScNup100)/TbNup158. Two localise exclusively to the macronuclei and carry GLFG repeats, while two are micronuclei-specific and carry NIFN repeats, likely functioning to differentially regulate MAC/MIC-specific transport [20].

## Pore membrane proteins

The MR is an integral structure of the NE [35], composed of POMs [9] and is possibly the least conserved NPC subcomplex (Figure 2). Only three POMs are candidates for a LECA origin: Ndc1 and Gp210, present broadly but partially lost from TSAR and excavates, with Gp210 also lost from algae and fungi [36]; and the more widely found POM121 [20,36]. All other POMs appear narrowly conserved, leading to structural deviation: Metazoan and fungal NPC ultrastructures are very different within the NE lumen, despite being comprised of structurally homologous domains of Gp210 or POM152, respectively [10,37–39], and no such similarities can be expected in algae or excavates lacking Gp210 [17,21]. Similarly, a particular function is difficult to assign to the core structural POMs. POM152, and the closed-mitosis fungai-specific POM34 [36], form a complex interaction network with NPC components but are non-essential in yeast [40]. However, Gp210 is not expressed in several tissues of mice and human primary fibroblasts [41,42] and reports are conflicting on the phenotype of Gp210 depletions in vertebrates and nematodes [43,44]. POM121 is dispensable in somatic human cell lines but critical in *Xenopus* embryos [44,45]. In *Tetrahymena*, POM121 is MAC-specific and distributes towards the NR side, but it is unknown if POM121 acts with MIC- and/or cytoplasmic side-specific TtPOM82 to alter OR stoichiometry [20]. Ndc1 depletion causes severe NPC assembly defects in metazoa and yeast, where it additionally functions in embedding spindle pole bodies into the NE [46–49]. Thus, the MR is a highly divergent structure and may lack a single defined architecture or function.

## The nuclear basket and mRNA export

**Figure 3.**
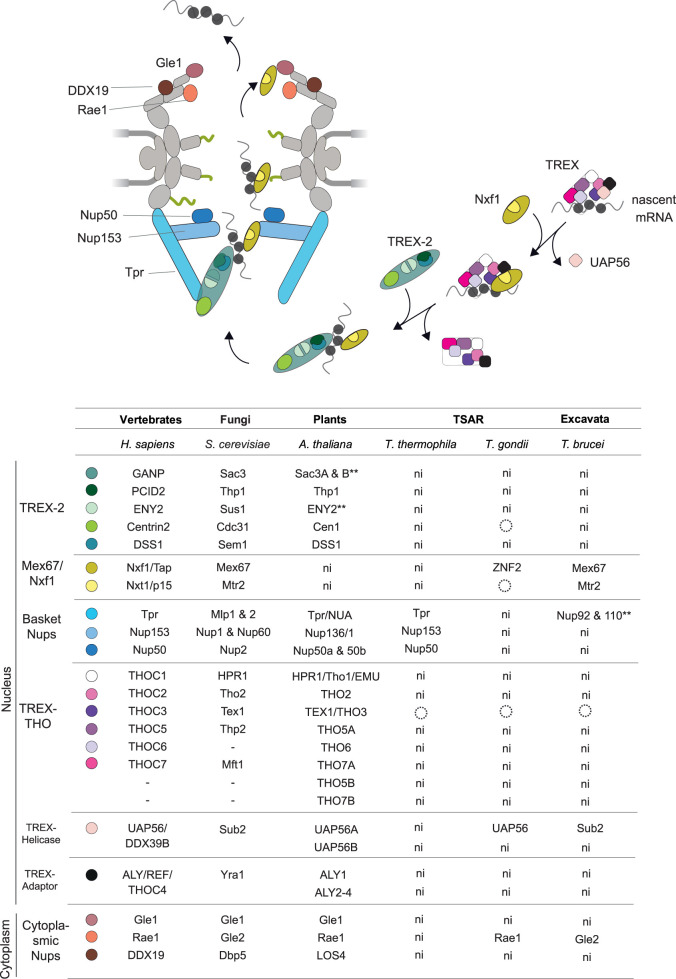
mRNA export machinery evolution. (**A**) Schematic summarising interactions and participants in the global RNA-independent mRNA export machinery. Nascent mRNA complexes with THO/TREX and after displacement of UAP56, export factor Nxf1 is recruited. mRNP is delivered to TREX-2 which facilitates the entry to the NPC. Nxf1 interacts with FG-repeats along the NPC and components at the cytoplasmic face catalyse the release of mRNA. (**B**) TREX and TREX-2 complexes, export factors, nuclear basket and cytoplasmic NPC components across taxa are summarised. Compared components are shown on the left, depicted with small coloured circles. All the experimental characterised components are written. With a dash, components are certainly absent, dotted circles, components identified *in silico*, but not experimentally characterised yet. Components marked with a double black star have been identified, but participation in mRNA export has not been proven. n.i. component not identified yet.

The NB in Metazoa consists of three highly conserved nucleoporins (Figures 2 and 3): Tpr, Nup50 and Nup153 [50], with yeast Mlp1/2, Nup2 and Nup1/Nup60 as respective homologues [51–53]. Tpr/Mlp proteins are involved in RNA biogenesis, spindle pole assembly, regulation of transcription, chromatin remodelling and mRNA export [54]. Although highly conserved within Metazoa, NB proteins suffered a significant diversification in other taxa, suggesting adaptations to organismal-specific roles. Tpr/Mlp homologues are more conserved than Nup153 and Nup50 and several organisms bear lineage-specific NB proteins [55–57].

Tpr, a coiled-coil homodimer, is the NB scaffold [58]. Nup153, a protein with RNA-binding properties [59], anchors to Tpr and the FG-repeats of Nup153 can reach into the NPC core [56,60]. It is unclear if Nup153 is essential for Tpr attachment to the NPC as data are conflicting [56,60–62]. It is likely that Nup153 is essential for Tpr recruitment during post-mitotic NPC assembly but not for anchoring Tpr already NPC localised, suggesting that an additional Tpr-binding site is present [63]. *A. thaliana* and *M. domestica* present divergent Tpr homologues (Tpr/NUA) [64–66] and also possess AtNup1/136, homologue of yeast Nup1/human Nup153 [18,67]. AtNup1 and AtTpr localise in the nuclear periphery during interphase with AtTpr localised in the vicinity of the spindle in pro-metaphase [66]. Significantly, C*. thermophilum* possesses two novel NB proteins, CtNup152 and CtNup56, which bear partial similarity to metazoan Nup50 and yeast Nup2 [14]. In trypanosomes, and conserved across kinetoplastids, TbNup92 and TbNup110 (coiled-coil proteins) constitute the NB and likely evolved independently from Mlp/Tpr [21].

The NB is a platform for the initial stages of RNA export, docking mRNPs and facilitating transport [10,68]. Tpr is crucial for recruiting TREX-2 complex components and hence efficient export of mRNA [61,63]. In yeast, Mlp1/Mlp2 provide docking sites for mRNPs and nuclear export adaptors, while Nup60 bears quality control capacity as Nup60 deletion causes Mlp1 mislocalisation and defects to pre-mRNAs retention [69]. In *Arabidopsis*, AtTpr and AtNup1 participate in polyA transcript export [18,64–67,70]. In *T. brucei*, NB proteins impact transcription of some RNA-binding proteins but precise roles remain undetermined [71].

## The Mex67–Mtr2 family

mRNA export pathways employ primarily the Nxf1–Nxt1 complex in mammals and their orthologs Mex67–Mtr2 in yeast. Sequence conservation between NFX1–NXT1 and Mex67–Mtr2 is low, albeit retaining functional complementarity [72,73]. Mex67–Mtr2 is the main mature mRNP carrier, an interaction essential for export [74,75]. Translocation of mRNPs across the NPC is mediated by interactions between Mex67–Mtr2 and FG-Nups [76–78]. Localisation of Mex67 at the NB is highly dependent on FG-repeats and deletions cause severe loss of Mex67 from the nuclear periphery [74]. Moreover, in humans, NXF1 presents additional tissue-specific isoforms [79] and ubiquitination regulates recruitment of Mex67 to nascent transcripts [80], adding further complexity to the regulation of mRNA export.

Mex67 has an RNA-recognition motif (RRM), a leucine-rich repeat (LRR), a nuclear transport factor 2-like (NTF2L) and ubiquitin-associated (UBA) domains [74,75,81]. The RRM binds interactors such as the TREX complex [82] and splicing factors [83,84]. Mtr2 also possesses an NTF2L domain, which contacts the Mex67 NTF2L region, forming a heterodimer. Although Mtr2 colocalises with the NPC, it does not directly contact the FG-repeats [76,77,85].

Orthologs of Mex67 and Mtr2 are both present in trypanosomes and essential for mRNA export [21,86–90]. Trypanosome Mex67 retains the LRR and the NTF2L domains, but has a divergent N-terminus with a CCCH-type Zn^2+^ finger replacing the typical RRM domain [21,91], while TbMtr2 also retains an NTF2L domain [21,91]. Additional TbMex67 paralogs are also present but their functions are unknown [90]. Importantly, TbMex67 interacts directly with Ran, suggesting that mRNA export is Ran-dependant and hence mechanistically distinct from animals and fungi [90,91]. In *T. gondii*, TgZnf2, a nuclear protein containing a C_2_H_2_ zinc finger, functions in mRNA export and cell cycle regulation and is highly conserved across Apicomplexa. TgZnf2 interacts with TgNtx1, a probable ortholog of Mtr2 [92]. In plants, Mex67 has not been found.

## Transcription-export complex 2

The TREX-2 complex has a crucial role in genome stability and transcription, providing an essential platform to recruit the mRNA processing machinery [93]; subunit deletions lead to nuclear mRNA accumulation [94]. TREX-2 is loaded onto mRNPs to aid translocation to the NPC and facilitates export by increasing the entry efficiency of mRNPs into the NPC transport channel [93,95]. In metazoa, TREX-2 interacts with the NPC in a highly stable manner [61].

In *S. cerevisiae* TREX-2 consists of six subunits (Sac3, Thp1, Sus1, Cdc31 and Sem1) with Sac3 acting as the scaffold [93]. In animals, the complex consists of a GANP scaffold, PCID2, two copies of ENY2, Centrin2 and DSS1 [96] as respective homologues. TREX-2, through FG-repeats in Sac3, interacts with the mRNP and Mex67/Mtr2, and through Sac3–Sus1–Cdc31 with Nup1 [96–99]. Homologues of TREX-2 components are present in *A. thaliana*, including Thp1, two Sac3 paralogs and orthologs of Cdc31 and Sem1 with physical interactions between them [67]. However, the Sus1 ortholog, AtENY2, is not a TREX-2 constituent [99,100] and rather is a component of the SAGA transcription complex [101]. As in other systems, depletion of Sac3 and Thp1 in *A. thaliana* causes mRNA [67] and miRNA accumulation [102]. To our knowledge, no components of TREX-2 have been functionally characterised in other taxa, with the possibility that they are present but too divergent to be identified by sequence comparison. In Apicomplexa, a potential Cdc31 has only been identified *in silico* [103].

## TREX

The TREX complex is co-transcriptionally recruited to nascent mRNAs and regulates splicing and export [104]. TREX consists of the multi-subunit THO complex, a conserved DEAD-box RNA helicase Sub2 and an export adaptor Yra1 (Figure 3), to which other components assemble. Sub2 has conserved functions promoting splicing, mRNA export and recruitment of Yra1. In the assembled TREX-mRNP complex, Sub2 together with Yra1 may load Mex67–Mtr2. Remarkably, although THO complex composition is clearly divergent between animals and fungi (Figure 3), recent data suggests that the overall tertiary structure, multimerization and flexibility of TREX are strikingly conserved [105]. Yra1 is essential in *S. cerevisiae* [106], but not metazoa [107,108] and overexpression in *A*. *thaliana* lacks an obvious phenotype [109]. This highlights that, although overall structure and mechanisms seem conserved, adaptations can contribute towards evolutionary context-dependent essentiality.

THO is conserved in plants but *A. thaliana* possesses additional THO and Yra1 paralogs (Figure 3), indicating complex diversity [110,111]. Trypanosomes have a Sub2 ortholog, an essential protein associated with mRNA transcription/processing sites and export [112], while in *T*. *gondii* a highly divergent ortholog has been characterised [113]. The remainder of the TREX complex in these organisms awaits discovery [114].

## Cytoplasmic filaments and mRNA export

At the cytoplasmic face of the NPC in yeast lies the Nup82 complex (Nup82, Nup159, Nsp1), a part of the export platform and attached to the cytoplasmic OR facing the central channel. Nup82 also helps Gle1, Dbp5 and Nup42 in organising the last stages of export. Dbp5 is an RNA-binding DEAD-box helices involved in transcription, mRNA export and termination of transcription [115,116]. During mRNA export, Dbp5 triggers remodelling of mRNAs emerging into the cytoplasm in the final export steps. Yeast Dbp5 is regulated by Gle1, an interaction stabilised by inositol-hexakisphosphate (IP_6_), which catalyses the release of RNA-binding proteins to ensure directional transport from the nucleus [117,118]. However, in humans IP_6_ binding may be dispensable [119], suggesting diverse mechanisms. Adding additional levels of regulation of mRNA export pathways, multiple Gle1 isoforms have been found [120].

In *Arabidopsis*, Gle1 is highly conserved [121], essential [122] and stimulates the Dbp5 homologue AtLOS4 [18,122]. Interestingly, in *Lotus japonicus*, Gle1 has evolved to promote a symbiotic relationship with mycorrhiza *Mesorhizobium loti* for symbiotic nitrogen fixation [123]. Trypanosomes lack orthologs of Gle1 and Dbp5 indicating a distinct mechanism [21] and consistent with simplified *trans*-splicing.

Gle2 (mammal/plant Rae1) is involved in mRNA export as inactivation/mislocalisation leads to nuclear accumulation of mRNA [124]. Interestingly, Gle2 is retained in trypanosomes and nucleomorph nuclei [21,125] (Figures 2 and 3), suggesting an ancient origin in the eukaryotic lineage and possibly a central role.

## Functional and genomic constraints moulding NPC evolution

The NPC is a remarkable example of co-evolution as mutations could result in complex effects produced by impacting many interacting partners [126,127]. Interestingly, Nups show different evolutionary rates which may reflect distinct evolutionary pressures [128]. Distinct functional constraints (Table 1) may influence this process and include protein–protein and protein–nucleic acid interactions [127,129]. Moreover, ubiquitously expressed proteins tend to evolve slower than tissue-specific proteins [129,130] and HsNup50, Tpr and Gp210 show differential mRNA and protein expression levels in different tissues, suggesting altered NPC composition between cell types [5,131].

**Table 1.** Functional and genomic constraints with potential impacts on NPC evolution

Constraint class	Constraint	Possible impact	Examples within the NPC
Functional	Structural environment of catalytic amino acids	Residues within the catalytic core are under greater pressure of being conserved. Residues are substituted in ways that the overall stabilities of structure are maintained [127,129]	Nups with enzymatic activity: Gle1, helicases Dpb5/DHX9. Autoproteolytic domain found in human Nup98/Nup96 (yeast 145N/145C) [135]
	Protein–protein interactions	Mould a complex network of protein : protein and subcomplex : subcomplex interactions. Restraints for the acceptance of amino acid substitutions based on interfaces contacts [127]	Subcomplexes within NPC. Different NPC stoichiometry across different tissues [5] Regulation of DHX9 helicase activity/distribution by Nup98 [136]
	Protein–nucleic acid interactions	Protein–nucleic acid recognition/interfaces tend to be highly conserved [127]	Rae1, Elys, Nup153 and ScNup157 capacity to bind nucleic acid [32,59,137]
Genomic	Gene expression: differential expression in tissues	Ubiquitously expressed proteins tend to evolve slower than tissue-specific proteins [129,130]	HsNup50, Tpr, Nup214, Aladin, Gp210, Pom121 and Nup37 levels (transcript and protein) are different in different tissues, suggesting rearrangements of NPC stoichiometry across cell types [5,131]
	Epigenetics: chromatin remodelling	Chromatin remodelling and epigenetic marks impact gene expression and therefore, protein evolution [126,130]	Nuclear basket roles in chromatin remodelling and epigenetic regulation (reviewed in [138])

An interesting further example is the autocatalytic domain of HsNup98/Sc145C. HsNup98 and HsNup96 are expressed as a single fusion protein which undergoes autoproteolytic processing [132], an event essential for localisation [133]. Interestingly, this mechanism is conserved in *S. cerevisiae* Nup145 [134], but absent from other organisms such as trypanosomes [21].

## Dynamic properties of the NPC

**Figure 4.**
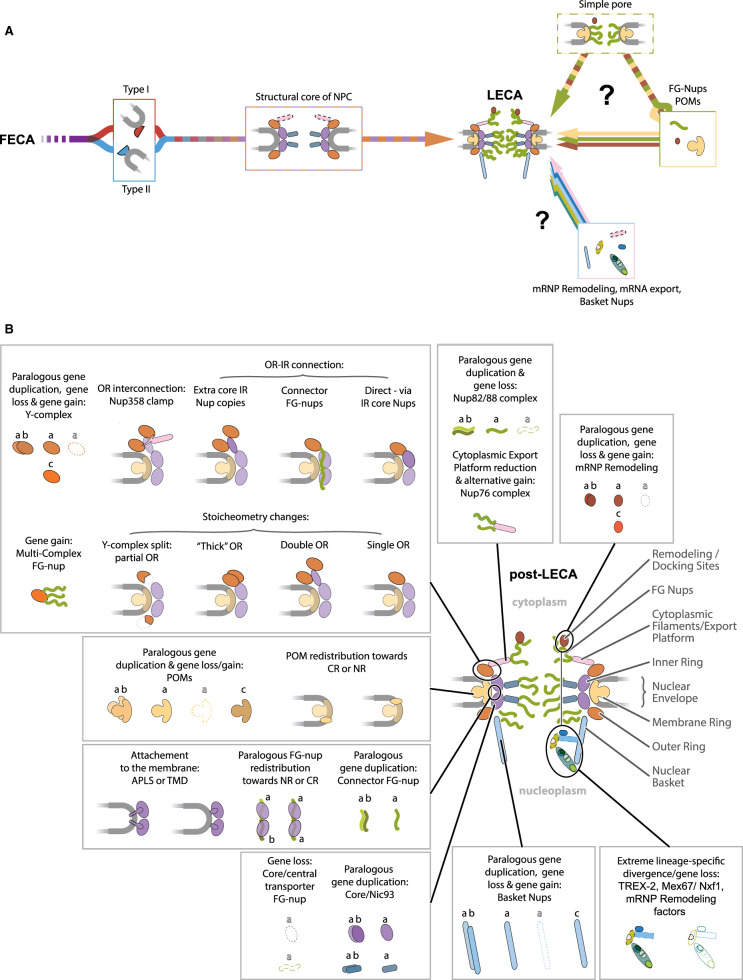
Outstanding questions in NPC evolution and known NPC diversifications. (**A**) While the structural core of LECA NPC — its inner and outer rings — can be convincingly traced to type I and type II coat proteins (left), it is yet unclear how the NPC acquired its other components — responsible for mRNA processing and general permselective function (right). As individual FG-nups/transport factor combinations were shown sufficient to execute partial per-selective function, and POMs — to form pores in lipid bilayers, the possibility arises that these components formed a separate simple pore before incorporation into NPC. (**B**) Summary of principal variations and evolutionary events in NPC architectures post-LECA. Principal architectural changes are named for each compartment. Principal evolutionary events are named and indicated. Paralogous duplication events indicated by a — a,b pairs with slight colour change. Gene loss — by dashed lines and letters. Gene gain — by a — c pairs and colour change. Changes in stoichiometry — shown schematically. Principal complex gains are named.

The inner and outer rings are the most conserved NPC subcomplexes and the α-solenoids, β-propellers and coiled-coil bundles within the ring nucleoporins account for the majority of mass density in cryo-EM structures. Conserved architectures trace NPC core evolution back to type I and type II coats (Figure 4A), but NPC modularity permits a variety of architectures (Figure 4B). It is important to understand that the NPC core is dynamic and there is a noticeable variation of the central channel diameter between 60 and 40 nm, respectively, depending on species and imaging method [7,10,17,135,136], representing the capacity of the NPC for constriction/dilation in response to environment [137], presence of transport factors/cargo [138–140], cell cycle stage [141] and mechanical force [142]. Such flexibility is thought to depend on several structural characteristics of the NPC. Firstly, within the central channel allosteric coupling exists between structured and intrinsically disordered FG-Nup domains in interactions with transport factors [139]. Furthermore, the lateral spoke interconnections within the inner ring are small [10] and contributing to the stability of the inner ring are ‘flexible connectors’, intrinsically disordered sequences interconnect NPC subcomplexes. For example, in *S. cerevisiae* these connectors are present in Nic96 that bridges Nup192 and the FG-Nups of the Nsp1 complex. Furthermore, Nup145N/Nup100/Nup116 interconnects inner and outer rings [16]. Notably, these interactions can be allosterically coupled by transport factors: Nup53 interaction with Kap121 destabilises Nic96 and Nup157 binding [140], potentially contributing to loosening of the inner ring and pore dilation to allow transport of cargo otherwise excluded from the NPC. Conservation of these interaction sites and allosteric interactions between animals and fungi [16,140] suggest such mechanisms are intrinsic to NPC function.

## The NPC during mitosis

Eukaryotes perform cell division by closed, open or semi-open mitosis [143]; in all cases, nucleoporins affect mitotic progression. Metazoans and higher plants (here represented by *H. Sapiens*, *A. thaliana* and *M. domestica*) undergo open mitosis that includes full breakdown of NE (NEBD) and NPC disassembly [143,144]. The remaining organisms, i.e. *C. reinhardtti*, *T. thermophila* and *T. brucei* perform close mitosis [145–147] which leaves the NE intact during cell division.

Although these models of nuclear division seem radically different, the mechanisms for disassembly of the NPC are strikingly similar, occurring in a highly synchronised manner. In animal cells, NPC disassembly occurs during NEBD and after phosphorylation of Nup98, while in *S. pombe* NPCs are gradually lost from the anaphase bridge connecting daughter nuclei. In open and closed division peripheral Nups are disassembled first, followed by the central scaffold and finally POMs [148–151]. If differences in NPC architecture influence the mitotic mode is unknown.

## Moonlighting NPC components

Moonlighting, or multi-functionality, is common amongst NPC and NPC-associated components and inevitably impacts selection constraints. NPC components are involved in a variety of pathways, located in additional compartments as individual nucleoporins or entire NPC subcomplexes. Sec13 (Y-complex) is a COPII component [152] and, together with Seh1 (also Y-complex) [153], are part of the SEA vacuolar complex. Significantly Nup62 and Nup188 are at mammalian centrosomes [154,155], and the entire mammalian Y-complex at kinetochores and, together with Seh1, recruit the chromosomal passenger complex [156–160]. Multiple nucleoporins promote chromatin decondensation, transcriptional activation [161–166] or epigenetic silencing [163] via direct localisation in the nucleoplasm or tethering chromosomal regions to assembled NPCs [167–173]. Very little is known in terms of conservation of moonlighting functions, although it is clear that trypanosomes do have similar processes, albeit with likely distinct evolutionary origins, suggesting possible convergence.

One emerging example of NPC component moonlighting is the ‘ciliary pore complex’ [174]. NPC components can localise at the base of the cilia and in human cells, these are the cytoplasmic filament Nup214, inner ring Nup35, Nup62 and Nup93, the outer ring Nup37 [174] and Nup75/Nup85 [175]. Additionally, Nup93 and multi-ring Nup188 [176] and, potentially, Nup205 [177] are thought to localise to the cilia base in *Xenopus*. These structures appear to support embryonic cilia formation and intraflagellar transport (IFT). Additional similarities between NPC and IFT, such as the ciliary localisation signal (CLS) likeness to NLS [178] and the requirement of CLS-recognising nuclear transport factor importin-β2 and a RanGTP/GDP gradient for the ciliary transport of several proteins [179,180], have added to the concept of a ciliary pore complex [174]. However, structural details and ubiquity remain to emerge and imaging of Nup188 in *Xenopus* shows structures incompatible with typical NPC organisation [174]. Among FG-nups, only Nup98 was found at the cilia [175] and ciliary transport appears insensitive to classic inhibitors of the NPC barrier [181]. The similarity in form and function between the NPC and ciliary pore complex is, therefore, unclear; however, the constraints applied by potential co-evolution between the nuclear and ciliary transport are intriguing. Interestingly, there is further mutual moonlighting between the NPC and cilia as centrin2, critical for centriole/centrosome, and thus cilia organisation [182], is also present in animal and fungal NPCs, contributing to mRNA and protein export [183].

## NPC evolution: a minimal pore?

The predictive and experimental structural analysis finds that the ∼30 proteins of the NPC core are composed predominantly of just eight structural fold types [25,26]. Of these, the three most frequent, α-solenoid, FG-repeat and β-propeller, account for >80% of residues. Notably, while sequence similarity is generally low, comparative genomics has identified over 20 nucleoporins from all three groups as conserved across all eukaryotic taxa [36] and thus likely represented in the LECA. Assuming that the NPC evolved incrementally with increased complexity, what forms did the NPC take during the transition from the FECA to the LECA (Figure 4A)? Phylogenetic and genetic analysis may be currently insufficient to resolve this question, but insights from other studies are valuable here.

Firstly, nucleomorphs, drastically reduced nuclei from red and green algae endosymbiotically absorbed into chlorarachniophytes and cryptophyceans [125], reveal a near-complete secondary reduction in algae NPC genes. The only NPC proteins that could participate in pore formation [184] are orthologs of Nup98 (in chlorarachniophytes) and Rae1 (in chlorarachniophytes and cryptophyceans) (Figure 2). However, there is no ultrastructural evidence for a pore-associated structure at the nucleomorph membrane and the retention of these subunits may indicate recruitment to other processes. Secondly, attempts to create an artificial pore, using truncated versions of yeast Nsp1 and Nup100 [185] or a designer ‘NupX’ [186], produced pores with selective permeability, suggesting minimal requirements to achieve gating. A self-assembling pore of just two POMs, Ndc1 and an FG-containing Pom121 in a lipid bilayer has also been achieved [187]. While these artificial or derived states almost definitely do not recapitulate the NPC during the FECA to LECA transition, they do indicate considerable potential for flexibility and great simplicity in mediating selective nucleocytoplasmic transport.

## Perspectives

- There are examples of clear divergence in NPC structure and likely functions across eukaryotes; more examples are needed to understand the basis for these changes.
- Mapping structure to function remains a major goal for NPC research, but understanding diversity and how these connect to biological aspects are also critical.
- Gaining deeper insight in the organisation of nuclear pores in nucleomorphs and into pore formation by POMs may aid reconstructions of the origins of permeability-selective NPCs.
- There is an urgent need to characterise mRNA export platforms (TREX, Mex67) and the manner in which these interface with the NPC, especially in non-canonical organisms.

## Abbreviations

CF: cytoplasmic filament
IFT: intraflagellar transport
LRR: leucine-rich repeat
MIC: micronucleus
MMB: membrane-binding domain
MR: membrane ring
NB: nuclear basket
NPC: nuclear pore complex
NTF2L: nuclear transport factor 2-like
POMs: pore membrane proteins
RRM: RNA-recognition motif

## References

[1] Fernandez-Martinez, J. and Rout, M.P. (2021) One ring to rule them all? Structural and functional diversity in the nuclear pore complex. Trends Biochem. Sci. 46, 595–607. 10.1016/j.tibs.2021.01.003 33563541 PMC8195821

[2] Field, M.C. and Rout, M.P. (2019) Pore timing: the evolutionary origins of the nucleus and nuclear pore complex. F1000Res 8, F1000 Faculty Rev-369 10.12688/f1000research.16402.1 PMC6449795 31001417

[3] Dacks, J.B. and Robinson, M.S. (2017) Outerwear through the ages: evolutionary cell biology of vesicle coats. Curr. Opin. Cell Biol. 47, 108–116 10.1016/j.ceb.2017.04.001 28622586

[4] Faini, M., Beck, R., Wieland, F.T. and Briggs, J.A. (2013) Vesicle coats: structure, function, and general principles of assembly. Trends Cell Biol. 23, 279–288 10.1016/j.tcb.2013.01.005 23414967

[5] Ori, A., Banterle, N., Iskar, M., Andres-Pons, A., Escher, C., Khanh Bui, H. et al. (2013) Cell type-specific nuclear pores: a case in point for context-dependent stoichiometry of molecular machines. Mol. Syst. Biol. 9, 648 10.1038/msb.2013.4 23511206 PMC3619942

[6] Bui, K.H., von Appen, A., DiGuilio, A.L., Ori, A., Sparks, L., Mackmull, M.T. et al. (2013) Integrated structural analysis of the human nuclear pore complex scaffold. Cell 155, 1233–1243 10.1016/j.cell.2013.10.055 24315095

[7] von Appen, A., Kosinski, J., Sparks, L., Ori, A., DiGuilio, A.L., Vollmer, B. et al. (2015) In situ structural analysis of the human nuclear pore complex. Nature 526, 526140–526143 10.1038/nature15381 PMC4886846 26416747

[8] Kosinski, J., Mosalaganti, S., von Appen, A., Teimer, R., DiGuilio, A.L., Wan, W. et al. (2016) Molecular architecture of the inner ring scaffold of the human nuclear pore complex. Science 352, 363–365 10.1126/science.aaf0643 27081072 PMC8926079

[9] Alber, F., Dokudovskaya, S., Veenhoff, L.M., Zhang, W., Kipper, J., Devos, D. et al. (2007) The molecular architecture of the nuclear pore complex. Nature 450, 695–701 10.1038/nature06405 18046406

[10] Kim, S.J., Fernandez-Martinez, J., Nudelman, I., Shi, Y., Zhang, W., Raveh, B. et al. (2018) Integrative structure and functional anatomy of a nuclear pore complex. Nature 555, 475–482 10.1038/nature26003 29539637 PMC6022767

[11] Kim, S.J., Fernandez-Martinez, J., Sampathkumar, P., Martel, A., Matsui, T., Tsuruta, H. et al. (2014) Integrative structure-function mapping of the nucleoporin Nup133 suggests a conserved mechanism for membrane anchoring of the nuclear pore complex. Mol. Cell. Proteom. 13, 2911–2926 10.1074/mcp.M114.040915 PMC4223481 25139911

[12] Asakawa, H., Yang, H.J., Yamamoto, T.G., Ohtsuki, C., Chikashige, Y., Sakata-Sogawa, K. et al. (2014) Characterization of nuclear pore complex components in fission yeast *Schizosaccharomyces pombe*. Nucleus 5, 149–162 10.4161/nucl.28487 24637836 PMC4049921

[13] Asakawa, H., Kojidani, T., Yang, H.J., Ohtsuki, C., Osakada, H., Matsuda, A. et al. (2019) Asymmetrical localization of Nup107-160 subcomplex components within the nuclear pore complex in fission yeast. PLoS Genet. 15, e1008061 10.1371/journal.pgen.1008061 31170156 PMC6553703

[14] Amlacher, S., Sarges, P., Flemming, D., van Noort, V., Kunze, R., Devos, D.P. et al. (2011) Insight into structure and assembly of the nuclear pore complex by utilizing the genome of a eukaryotic thermophile. Cell 146, 277–289 10.1016/j.cell.2011.06.039 21784248

[15] Thierbach, K., von Appen, A., Thoms, M., Beck, M., Flemming, D. and Hurt, E. (2013) Protein interfaces of the conserved Nup84 complex from *Chaetomium thermophilum* shown by crosslinking mass spectrometry and electron microscopy. Structure 21, 1672–1682 10.1016/j.str.2013.07.004 23954503

[16] Fischer, J., Teimer, R., Amlacher, S., Kunze, R. and Hurt, E. (2015) Linker Nups connect the nuclear pore complex inner ring with the outer ring and transport channel. Nat. Struct. Mol. Biol. 22, 774–781 10.1038/nsmb.3084 26344569

[17] Mosalaganti, S., Kosinski, J., Albert, S., Schaffer, M., Strenkert, D., Salome, P.A. et al. (2018) In situ architecture of the algal nuclear pore complex. Nat. Commun. 9, 2361 10.1038/s41467-018-04739-y 29915221 PMC6006428

[18] Tamura, K., Fukao, Y., Iwamoto, M., Haraguchi, T. and Hara-Nishimura, I. (2010) Identification and characterization of nuclear pore complex components in *Arabidopsis thaliana*. Plant Cel 22, 4084–4097 10.1105/tpc.110.079947 PMC3027183 21189294

[19] Zhang, C., An, N., Jia, P., Zhang, W., Liang, J., Zhang, X. et al. (2020) Genomic identification and expression analysis of nuclear pore proteins in *Malus domestica*. Sci. Rep. 10, 17426 10.1038/s41598-020-74171-0 33060661 PMC7566457

[20] Iwamoto, M., Osakada, H., Mori, C., Fukuda, Y., Nagao, K., Obuse, C. et al. (2017) Compositionally distinct nuclear pore complexes of functionally distinct dimorphic nuclei in the ciliate Tetrahymena. J. Cell Sci. 130, 1822–1834 10.1242/jcs.199398 28386019 PMC5450191

[21] Obado, S.O., Brillantes, M., Uryu, K., Zhang, W., Ketaren, N.E., Chait, B.T. et al. (2016) Interactome mapping reveals the evolutionary history of the nuclear pore complex. PLoS Biol. 14, e1002365 10.1371/journal.pbio.1002365 26891179 PMC4758718

[22] Drin, G., Casella, J.F., Gautier, R., Boehmer, T., Schwartz, T.U. and Antonny, B. (2007) A general amphipathic alpha-helical motif for sensing membrane curvature. Nat. Struct. Mol. Biol. 14, 138–146 10.1038/nsmb1194 17220896

[23] Shi, Y., Fernandez-Martinez, J., Tjioe, E., Pellarin, R., Kim, S.J., Williams, R. et al. (2014) Structural characterization by cross-linking reveals the detailed architecture of a coatomer-related heptameric module from the nuclear pore complex. Mol. Cell. Proteom. 13, 2927–2943 10.1074/mcp.M114.041673 PMC4223482 25161197

[24] Nordeen, S.A., Turman, D.L. and Schwartz, T.U. (2020) Yeast Nup84-Nup133 complex structure details flexibility and reveals conservation of the membrane anchoring ALPS motif. Nat. Commun. 11, 6060 10.1038/s41467-020-19885-5 33247142 PMC7695694

[25] Devos, D., Dokudovskaya, S., Alber, F., Williams, R., Chait, B.T., Sali, A. et al. (2004) Components of coated vesicles and nuclear pore complexes share a common molecular architecture. PLoS Biol. 2, e380 10.1371/journal.pbio.0020380 15523559 PMC524472

[26] Devos, D., Dokudovskaya, S., Williams, R., Alber, F., Eswar, N., Chait, B.T. et al. (2006) Simple fold composition and modular architecture of the nuclear pore complex. Proc. Natl Acad. Sci. U.S.A. 103, 2172–2177 10.1073/pnas.0506345103 16461911 PMC1413685

[27] DeGrasse, J.A., DuBois, K.N., Devos, D., Siegel, T.N., Sali, A., Field, M.C. et al. (2009) Evidence for a shared nuclear pore complex architecture that is conserved from the last common eukaryotic ancestor. Mol. Cell. Proteom. 8, 2119–2130 10.1074/mcp.M900038-MCP200 PMC2742445 19525551

[28] Lutzmann, M., Kunze, R., Buerer, A., Aebi, U. and Hurt, E. (2002) Modular self-assembly of a Y-shaped multiprotein complex from seven nucleoporins. EMBO J. 21, 387–397 10.1093/emboj/21.3.387 11823431 PMC125826

[29] Huang, G., Zhang, Y., Zhu, X., Zeng, C., Wang, Q., Zhou, Q. et al. (2020) Structure of the cytoplasmic ring of the *Xenopus laevis* nuclear pore complex by cryo-electron microscopy single particle analysis. Cell Res. 30, 520–531 10.1038/s41422-020-0319-4 32376910 PMC7264146

[30] Kimura, N., Takizawa, M., Okita, K., Natori, O., Igarashi, K., Ueno, M. et al. (2002) Identification of a novel transcription factor, ELYS, expressed predominantly in mouse foetal haematopoietic tissues. Genes Cells 7, 435–446 10.1046/j.1365-2443.2002.00529.x 11952839

[31] Rasala, B.A., Orjalo, A.V., Shen, Z., Briggs, S. and Forbes, D.J. (2006) ELYS is a dual nucleoporin/kinetochore protein required for nuclear pore assembly and proper cell division. Proc. Natl Acad. Sci. U.S.A. 103, 17801–17806 10.1073/pnas.0608484103 17098863 PMC1635652

[32] Rasala, B.A., Ramos, C., Harel, A. and Forbes, D.J. (2008) Capture of AT-rich chromatin by ELYS recruits POM121 and NDC1 to initiate nuclear pore assembly. Mol. Biol. Cell 19, 3982–3996 10.1091/mbc.e08-01-0012 18596237 PMC2526682

[33] Padilla-Mejia, N.E., Makarov, A.A., Barlow, L.D., Butterfield, E.R. and Field, M.C. (2021) Evolution and diversification of the nuclear envelope. Nucleus 12, 21–41 10.1080/19491034.2021.1874135 33435791 PMC7889174

[34] Koreny, L. and Field, M.C. (2016) Ancient eukaryotic origin and evolutionary plasticity of nuclear lamina. Genome Biol. Evol. 8, 2663–2671 10.1093/gbe/evw087 27189989 PMC5630835

[35] Akey, C.W. and Radermacher, M. (1993) Architecture of the Xenopus nuclear pore complex revealed by three-dimensional cryo-electron microscopy. J. Cell Biol. 122, 1–19 10.1083/jcb.122.1.1 8314837 PMC2119598

[36] Neumann, N., Lundin, D. and Poole, A.M. (2010) Comparative genomic evidence for a complete nuclear pore complex in the last eukaryotic common ancestor. PLoS One 5, e13241 10.1371/journal.pone.0013241 20949036 PMC2951903

[37] Upla, P., Kim, S.J., Sampathkumar, P., Dutta, K., Cahill, S.M., Chemmama, I.E. et al. (2017) Molecular architecture of the major membrane ring component of the nuclear pore complex. Structure 25, 434–445 10.1016/j.str.2017.01.006 28162953 PMC5342941

[38] Hao, Q., Zhang, B., Yuan, K., Shi, H. and Blobel, G. (2018) Electron microscopy of Chaetomium pom152 shows the assembly of ten-bead string. Cell Discov. 4, 56 10.1038/s41421-018-0057-7 30245846 PMC6141588

[39] Zhang, Y., Li, S., Zeng, C., Huang, G., Zhu, X., Wang, Q. et al. (2020) Molecular architecture of the luminal ring of the *Xenopus laevis* nuclear pore complex. Cell Res. 30, 532–540 10.1038/s41422-020-0320-y 32367042 PMC7264284

[40] Wozniak, R.W., Blobel, G. and Rout, M.P. (1994) POM152 is an integral protein of the pore membrane domain of the yeast nuclear envelope. J. Cell Biol. 125, 31–42 10.1083/jcb.125.1.31 8138573 PMC2120016

[41] Eriksson, C., Rustum, C. and Hallberg, E. (2004) Dynamic properties of nuclear pore complex proteins in gp210 deficient cells. FEBS Lett. 572, 261–265 10.1016/j.febslet.2004.07.044 15304359

[42] Olsson, M., Scheele, S. and Ekblom, P. (2004) Limited expression of nuclear pore membrane glycoprotein 210 in cell lines and tissues suggests cell-type specific nuclear pores in metazoans. Exp. Cell Res. 292, 359–370 10.1016/j.yexcr.2003.09.014 14697343

[43] Cohen, M., Feinstein, N., Wilson, K.L. and Gruenbaum, Y. (2003) Nuclear pore protein gp210 is essential for viability in HeLa cells and *Caenorhabditis elegans*. Mol. Biol. Cell 14, 4230–4237 10.1091/mbc.e03-04-0260 14517331 PMC207014

[44] Stavru, F., Nautrup-Pedersen, G., Cordes, V.C. and Gorlich, D. (2006) Nuclear pore complex assembly and maintenance in POM121- and gp210-deficient cells. J. Cell Biol. 173, 477–483 10.1083/jcb.200601002 16702234 PMC2063858

[45] Antonin, W., Franz, C., Haselmann, U., Antony, C. and Mattaj, I.W. (2005) The integral membrane nucleoporin pom121 functionally links nuclear pore complex assembly and nuclear envelope formation. Mol. Cell 17, 83–92 10.1016/j.molcel.2004.12.010 15629719

[46] Winey, M., Hoyt, M.A., Chan, C., Goetsch, L., Botstein, D. and Byers, B. (1993) NDC1: a nuclear periphery component required for yeast spindle pole body duplication. J. Cell Biol. 122, 743–751 10.1083/jcb.122.4.743 8349727 PMC2119589

[47] West, R.R., Vaisberg, E.V., Ding, R., Nurse, P. and McIntosh, J.R. (1998) Cut11(+): a gene required for cell cycle-dependent spindle pole body anchoring in the nuclear envelope and bipolar spindle formation in *Schizosaccharomyces pombe*. Mol. Biol. Cell 9, 2839–2855 10.1091/mbc.9.10.2839 9763447 PMC25557

[48] Eisenhardt, N., Redolfi, J. and Antonin, W. (2014) Interaction of Nup53 with Ndc1 and Nup155 is required for nuclear pore complex assembly. J. Cell Sci. 127, 908–921 10.1242/jcs.141739 24363447

[49] Stavru, F., Hulsmann, B.B., Spang, A., Hartmann, E., Cordes, V.C. and Gorlich, D. (2006) NDC1: a crucial membrane-integral nucleoporin of metazoan nuclear pore complexes. J. Cell Biol. 173, 509–519 10.1083/jcb.200601001 16702233 PMC2063861

[50] Frosst, P., Guan, T., Subauste, C., Hahn, K. and Gerace, L. (2002) Tpr is localized within the nuclear basket of the pore complex and has a role in nuclear protein export. J. Cell Biol. 156, 617–630 10.1083/jcb.200106046 11839768 PMC2174070

[51] Ashkenazy-Titelman, A., Shav-Tal, Y. and Kehlenbach, R.H. (2020) Into the basket and beyond: the journey of mRNA through the nuclear pore complex. Biochem. J. 477, 23–44 10.1042/BCJ20190132 31913454

[52] Strambio-de-Castillia, C., Blobel, G. and Rout, M.P. (1999) Proteins connecting the nuclear pore complex with the nuclear interior. J. Cell Biol. 144, 839–855 10.1083/jcb.144.5.839 10085285 PMC2148185

[53] Kosova, B., Pante, N., Rollenhagen, C., Podtelejnikov, A., Mann, M., Aebi, U. et al. (2000) Mlp2p, a component of nuclear pore attached intranuclear filaments, associates with nic96p. J. Biol. Chem. 275, 343–350 10.1074/jbc.275.1.343 10617624

[54] Niepel, M., Molloy, K.R., Williams, R., Farr, J.C., Meinema, A.C., Vecchietti, N. et al. (2013) The nuclear basket proteins Mlp1p and Mlp2p are part of a dynamic interactome including Esc1p and the proteasome. Mol. Biol. Cell 24, 3920–3938 10.1091/mbc.e13-07-0412 24152732 PMC3861087

[55] Makise, M., Mackay, D.R., Elgort, S., Shankaran, S.S., Adam, S.A. and Ullman, K.S. (2012) The Nup153-Nup50 protein interface and its role in nuclear import. J. Biol. Chem. 287, 38515–38522 10.1074/jbc.M112.378893 23007389 PMC3493896

[56] Duheron, V., Chatel, G., Sauder, U., Oliveri, V. and Fahrenkrog, B. (2014) Structural characterization of altered nucleoporin Nup153 expression in human cells by thin-section electron microscopy. Nucleus 5, 601–612 10.4161/19491034.2014.990853 25485891 PMC4615255

[57] Lin, D.H. and Hoelz, A. (2019) The structure of the nuclear pore complex (an update). Annu. Rev. Biochem. 88, 725–783 10.1146/annurev-biochem-062917-011901 30883195 PMC6588426

[58] Krull, S., Thyberg, J., Bjorkroth, B., Rackwitz, H.R. and Cordes, V.C. (2004) Nucleoporins as components of the nuclear pore complex core structure and Tpr as the architectural element of the nuclear basket. Mol. Biol. Cell 15, 4261–4277 10.1091/mbc.e04-03-0165 15229283 PMC515357

[59] Ball, J.R., Dimaano, C., Bilak, A., Kurchan, E., Zundel, M.T. and Ullman, K.S. (2007) Sequence preference in RNA recognition by the nucleoporin Nup153. J. Biol. Chem. 282, 8734–8740 10.1074/jbc.M608477200 17242408

[60] Soop, T., Ivarsson, B., Bjorkroth, B., Fomproix, N., Masich, S., Cordes, V.C. et al. (2005) Nup153 affects entry of messenger and ribosomal ribonucleoproteins into the nuclear basket during export. Mol. Biol. Cell 16, 5610–5620 10.1091/mbc.e05-08-0715 16195343 PMC1289406

[61] Umlauf, D., Bonnet, J., Waharte, F., Fournier, M., Stierle, M., Fischer, B. et al. (2013) The human TREX-2 complex is stably associated with the nuclear pore basket. J. Cell Sci. 126(Pt 12), 2656–2667 10.1242/jcs.118000 23591820

[62] Walther, T.C., Fornerod, M., Pickersgill, H., Goldberg, M., Allen, T.D. and Mattaj, I.W. (2001) The nucleoporin Nup153 is required for nuclear pore basket formation, nuclear pore complex anchoring and import of a subset of nuclear proteins. EMBO J. 20, 5703–5714 10.1093/emboj/20.20.5703 11598013 PMC125666

[63] Aksenova, V., Smith, A., Lee, H., Bhat, P., Esnault, C., Chen, S. et al. (2020) Nucleoporin TPR is an integral component of the TREX-2 mRNA export pathway. Nat. Commun. 11, 4577 10.1038/s41467-020-18266-2 32917881 PMC7486939

[64] Zhang, B., You, C., Zhang, Y., Zeng, L., Hu, J., Zhao, M. et al. (2020) Linking key steps of microRNA biogenesis by TREX-2 and the nuclear pore complex in Arabidopsis. Nat. Plants 6, 957–969 10.1038/s41477-020-0726-z 32690891 PMC7426256

[65] Jacob, Y., Mongkolsiriwatana, C., Veley, K.M., Kim, S.Y. and Michaels, S.D. (2007) The nuclear pore protein AtTPR is required for RNA homeostasis, flowering time, and auxin signaling. Plant Physiol. 144, 1383–1390 10.1104/pp.107.100735 17535820 PMC1914142

[66] Xu, X.M., Rose, A., Muthuswamy, S., Jeong, S.Y., Venkatakrishnan, S., Zhao, Q. et al. (2007) NUCLEAR PORE ANCHOR, the Arabidopsis homolog of Tpr/Mlp1/Mlp2/megator, is involved in mRNA export and SUMO homeostasis and affects diverse aspects of plant development. Plant Cell 19, 1537–1548 10.1105/tpc.106.049239 17513499 PMC1913724

[67] Lu, Q., Tang, X., Tian, G., Wang, F., Liu, K., Nguyen, V. et al. (2010) Arabidopsis homolog of the yeast TREX-2 mRNA export complex: components and anchoring nucleoporin. Plant J. 61, 259–270 10.1111/j.1365-313X.2009.04048.x 19843313

[68] Lee, E.S., Wolf, E.J., Ihn, S.S.J., Smith, H.W., Emili, A. and Palazzo, A.F. (2020) TPR is required for the efficient nuclear export of mRNAs and lncRNAs from short and intron-poor genes. Nucleic Acids Res. 48, 11645–11663 10.1093/nar/gkaa919 33091126 PMC7672458

[69] Galy, V., Gadal, O., Fromont-Racine, M., Romano, A., Jacquier, A. and Nehrbass, U. (2004) Nuclear retention of unspliced mRNAs in yeast is mediated by perinuclear Mlp1. Cell 116, 63–73 10.1016/S0092-8674(03)01026-2 14718167

[70] Bi, X., Cheng, Y.J., Hu, B., Ma, X., Wu, R., Wang, J.W. et al. (2017) Nonrandom domain organization of the Arabidopsis genome at the nuclear periphery. Genome Res. 27, 1162–1173 10.1101/gr.215186.116 28385710 PMC5495068

[71] Holden, J.M., Koreny, L., Obado, S., Ratushny, A.V., Chen, W.M., Chiang, J.H. et al. (2014) Nuclear pore complex evolution: a trypanosome Mlp analogue functions in chromosomal segregation but lacks transcriptional barrier activity. Mol. Biol. Cell 25, 1421–1436 10.1091/mbc.e13-12-0750 24600046 PMC4004592

[72] Katahira, J., Strasser, K., Podtelejnikov, A., Mann, M., Jung, J.U. and Hurt, E. (1999) The Mex67p-mediated nuclear mRNA export pathway is conserved from yeast to human. EMBO J. 18, 2593–2609 10.1093/emboj/18.9.2593 10228171 PMC1171339

[73] Chen, S., Wang, R., Zheng, D., Zhang, H., Chang, X., Wang, K. et al. (2019) The mRNA export receptor NXF1 coordinates transcriptional dynamics, alternative polyadenylation, and mrna export. Mol. Cell 74, 118–131.e7 10.1016/j.molcel.2019.01.026 30819645 PMC6450743

[74] Aibara, S., Katahira, J., Valkov, E. and Stewart, M. (2015) The principal mRNA nuclear export factor NXF1:NXT1 forms a symmetric binding platform that facilitates export of retroviral CTE-RNA. Nucleic Acids Res. 43, 1883–1893 10.1093/nar/gkv032 25628361 PMC4330390

[75] Aibara, S., Valkov, E., Lamers, M.H., Dimitrova, L., Hurt, E. and Stewart, M. (2015) Structural characterization of the principal mRNA-export factor Mex67-Mtr2 from *Chaetomium thermophilum*. Acta Crystallogr. F Struct. Biol. Commun. 71(Pt 7), 876–888 10.1107/S2053230X15008766 26144233 PMC4498709

[76] Ben-Yishay, R., Mor, A., Shraga, A., Ashkenazy-Titelman, A., Kinor, N., Schwed-Gross, A. et al. (2019) Imaging within single NPCs reveals NXF1's role in mRNA export on the cytoplasmic side of the pore. J. Cell Biol. 218, 2962–2981 10.1083/jcb.201901127 31375530 PMC6719458

[77] Derrer, C.P., Mancini, R., Vallotton, P., Huet, S., Weis, K. and Dultz, E. (2019) The RNA export factor Mex67 functions as a mobile nucleoporin. J. Cell Biol. 218, 3967–3976 10.1083/jcb.201909028 31753862 PMC6891080

[78] Stewart, M. (2010) Nuclear export of mRNA. Trends Biochem. Sci. 35, 609–617 10.1016/j.tibs.2010.07.001 20719516

[79] Li, Y., Bor, Y.C., Fitzgerald, M.P., Lee, K.S., Rekosh, D. and Hammarskjold, M.L. (2016) An NXF1 mRNA with a retained intron is expressed in hippocampal and neocortical neurons and is translated into a protein that functions as an Nxf1 cofactor. Mol. Biol. Cell 27, 3903–3912 10.1091/mbc.E16-07-0515 27708137 PMC5170612

[80] Eyboulet, F., Jeronimo, C., Cote, J. and Robert, F. (2020) The deubiquitylase Ubp15 couples transcription to mRNA export. eLife 9, e61264 10.7554/eLife.61264 33226341 PMC7682988

[81] Aibara, S., Valkov, E., Lamers, M. and Stewart, M. (2015) Domain organization within the nuclear export factor Mex67:Mtr2 generates an extended mRNA binding surface. Nucleic Acids Res. 43, 1927–1936 10.1093/nar/gkv030 25618852 PMC4330389

[82] Viphakone, N., Hautbergue, G.M., Walsh, M., Chang, C.T., Holland, A., Folco, E.G. et al. (2012) TREX exposes the RNA-binding domain of Nxf1 to enable mRNA export. Nat. Commun. 3, 1006 10.1038/ncomms2005 22893130 PMC3654228

[83] Huang, Y., Gattoni, R., Stevenin, J. and Steitz, J.A. (2003) SR splicing factors serve as adapter proteins for TAP-dependent mRNA export. Mol. Cell 11, 837–843 10.1016/S1097-2765(03)00089-3 12667464

[84] Muller-McNicoll, M., Botti, V., de Jesus Domingues, A.M., Brandl, H., Schwich, O.D., Steiner, M.C. et al. (2016) SR proteins are NXF1 adaptors that link alternative RNA processing to mRNA export. Genes Dev. 30, 553–566 10.1101/gad.276477.115 26944680 PMC4782049

[85] Fribourg, S., Braun, I.C., Izaurralde, E. and Conti, E. (2001) Structural basis for the recognition of a nucleoporin FG repeat by the NTF2-like domain of the TAP/p15 mRNA nuclear export factor. Mol. Cell 8, 645–656 10.1016/S1097-2765(01)00348-3 11583626

[86] Kramer, S., Kimblin, N.C. and Carrington, M. (2010) Genome-wide in silico screen for CCCH-type zinc finger proteins of *Trypanosoma brucei*, *Trypanosoma cruzi* and *Leishmania major*. BMC Genomics 11, 283 10.1186/1471-2164-11-283 20444260 PMC2873481

[87] Dean, S., Sunter, J.D. and Wheeler, R.J. (2017) Tryptag.org: a trypanosome genome-wide protein localisation resource. Trends Parasitol. 33, 80–82 10.1016/j.pt.2016.10.009 27863903 PMC5270239

[88] Aslett, M., Aurrecoechea, C., Berriman, M., Brestelli, J., Brunk, B.P., Carrington, M. et al. (2010) TriTrypDB: a functional genomic resource for the Trypanosomatidae. Nucleic Acids Res. 38, D457–D462 10.1093/nar/gkp851 19843604 PMC2808979

[89] Dostalova, A., Kaser, S., Cristodero, M. and Schimanski, B. (2013) The nuclear mRNA export receptor Mex67-Mtr2 of *Trypanosoma brucei* contains a unique and essential zinc finger motif. Mol. Microbiol. 88, 728–739 10.1111/mmi.12217 23560737

[90] Schwede, A., Manful, T., Jha, B.A., Helbig, C., Bercovich, N., Stewart, M. et al. (2009) The role of deadenylation in the degradation of unstable mRNAs in trypanosomes. Nucleic Acids Res. 37, 5511–5528 10.1093/nar/gkp571 19596809 PMC2760810

[91] Rink, C. and Williams, N. (2019) Unique interactions of the nuclear export receptors TbMex67 and TbMtr2 with components of the 5S ribonuclear particle in *Trypanosoma brucei*. mSphere 4, e00471-19 10.1128/mSphere.00471-19 31413174 PMC6695518

[92] Gissot, M., Hovasse, A., Chaloin, L., Schaeffer-Reiss, C., Van Dorsselaer, A. and Tomavo, S. (2017) An evolutionary conserved zinc finger protein is involved in *Toxoplasma gondii* mRNA nuclear export. Cell Microbiol. 19, e12644 10.1111/cmi.12644 27385072

[93] Stewart, M. (2019) Structure and function of the TREX-2 complex. Subcell Biochem. 93, 461–470 10.1007/978-3-030-28151-9_15 31939161

[94] Jani, D., Lutz, S., Hurt, E., Laskey, R.A., Stewart, M. and Wickramasinghe, V.O. (2012) Functional and structural characterization of the mammalian TREX-2 complex that links transcription with nuclear messenger RNA export. Nucleic Acids Res. 40, 4562–4573 10.1093/nar/gks059 22307388 PMC3378895

[95] Dimitrova, L., Valkov, E., Aibara, S., Flemming, D., McLaughlin, S.H., Hurt, E. et al. (2015) Structural characterization of the *Chaetomium thermophilum* TREX-2 complex and its interaction with the mRNA nuclear export factor Mex67:Mtr2. Structure 23, 1246–1257 10.1016/j.str.2015.05.002 26051714 PMC4509546

[96] Jani, D., Valkov, E. and Stewart, M. (2014) Structural basis for binding the TREX2 complex to nuclear pores, GAL1 localisation and mRNA export. Nucleic Acids Res. 42, 6686–6697 10.1093/nar/gku252 24705649 PMC4041426

[97] Ellisdon, A.M., Dimitrova, L., Hurt, E. and Stewart, M. (2012) Structural basis for the assembly and nucleic acid binding of the TREX-2 transcription-export complex. Nat. Struct. Mol. Biol. 19, 328–336 10.1038/nsmb.2235 22343721 PMC3303126

[98] Gordon, J.M.B., Aibara, S. and Stewart, M. (2017) Structure of the Sac3 RNA-binding M-region in the *Saccharomyces cerevisiae* TREX-2 complex. Nucleic Acids Res. 45, 5577–5585 10.1093/nar/gkx158 28334829 PMC5435946

[99] Jani, D., Lutz, S., Marshall, N.J., Fischer, T., Kohler, A., Ellisdon, A.M. et al. (2009) Sus1, Cdc31, and the Sac3 CID region form a conserved interaction platform that promotes nuclear pore association and mRNA export. Mol. Cell 33, 727–737 10.1016/j.molcel.2009.01.033 19328066 PMC2726291

[100] Sorensen, B.B., Ehrnsberger, H.F., Esposito, S., Pfab, A., Bruckmann, A., Hauptmann, J. et al. (2017) The Arabidopsis THO/TREX component TEX1 functionally interacts with MOS11 and modulates mRNA export and alternative splicing events. Plant Mol. Biol. 93, 283–298 10.1007/s11103-016-0561-9 28004241

[101] Pfab, A., Bruckmann, A., Nazet, J., Merkl, R. and Grasser, K.D. (2018) The adaptor protein ENY2 is a component of the deubiquitination module of the Arabidopsis SAGA transcriptional co-activator complex but not of the TREX-2 complex. J. Mol. Biol. 430, 1479–1494 10.1016/j.jmb.2018.03.018 29588169

[102] Yang, Y., La, H., Tang, K., Miki, D., Yang, L., Wang, B. et al. (2017) SAC3B, a central component of the mRNA export complex TREX-2, is required for prevention of epigenetic gene silencing in Arabidopsis. Nucleic Acids Res. 45, 181–197 10.1093/nar/gkw850 27672037 PMC5224508

[103] Avila, A.R., Cabezas-Cruz, A. and Gissot, M. (2018) mRNA export in the apicomplexan parasite *Toxoplasma gondii*: emerging divergent components of a crucial pathway. Parasit Vectors 11, 62 10.1186/s13071-018-2648-4 29370868 PMC5785795

[104] Meinel, D.M., Burkert-Kautzsch, C., Kieser, A., O'Duibhir, E., Siebert, M., Mayer, A. et al. (2013) Recruitment of TREX to the transcription machinery by its direct binding to the phospho-CTD of RNA polymerase II. PLoS Genet. 9, e1003914 10.1371/journal.pgen.1003914 24244187 PMC3828145

[105] Puhringer, T., Hohmann, U., Fin, L., Pacheco-Fiallos, B., Schellhaas, U., Brennecke, J. et al. (2020) Structure of the human core transcription-export complex reveals a hub for multivalent interactions. eLife 9, e61503 10.7554/eLife.61503 33191911 PMC7744094

[106] Portman, D.S., O'Connor, J.P. and Dreyfuss, G. (1997) YRA1, an essential *Saccharomyces cerevisiae* gene, encodes a novel nuclear protein with RNA annealing activity. RNA 3, 527–537 PMID: 9149233 PMC1369502

[107] Longman, D., Johnstone, I.L. and Caceres, J.F. (2003) The Ref/Aly proteins are dispensable for mRNA export and development in *Caenorhabditis elegans*. RNA 9, 881–891 10.1261/rna.5420503 12810921 PMC1370454

[108] Gatfield, D. and Izaurralde, E. (2002) REF1/Aly and the additional exon junction complex proteins are dispensable for nuclear mRNA export. J. Cell Biol. 159, 579–588 10.1083/jcb.200207128 12438415 PMC2173090

[109] Kammel, C., Thomaier, M., Sorensen, B.B., Schubert, T., Langst, G., Grasser, M. et al. (2013) Arabidopsis DEAD-box RNA helicase UAP56 interacts with both RNA and DNA as well as with mRNA export factors. PLoS One 8, e60644 10.1371/journal.pone.0060644 23555998 PMC3608606

[110] Yelina, N.E., Smith, L.M., Jones, A.M., Patel, K., Kelly, K.A. and Baulcombe, D.C. (2010) Putative Arabidopsis THO/TREX mRNA export complex is involved in transgene and endogenous siRNA biosynthesis. Proc. Natl Acad. Sci. U.S.A. 107, 13948–13953 10.1073/pnas.0911341107 20634427 PMC2922225

[111] Pfaff, C., Ehrnsberger, H.F., Flores-Tornero, M., Sorensen, B.B., Schubert, T., Langst, G. et al. (2018) ALY RNA-binding proteins are required for nucleocytosolic mRNA transport and modulate plant growth and development. Plant Physiol. 177, 226–240 10.1104/pp.18.00173 29540591 PMC5933122

[112] Serpeloni, M., Moraes, C.B., Muniz, J.R., Motta, M.C., Ramos, A.S., Kessler, R.L. et al. (2011) An essential nuclear protein in trypanosomes is a component of mRNA transcription/export pathway. PLoS One 6, e20730 10.1371/journal.pone.0020730 21687672 PMC3110772

[113] Serpeloni, M., Jimenez-Ruiz, E., Vidal, N.M., Kroeber, C., Andenmatten, N., Lemgruber, L. et al. (2016) UAP56 is a conserved crucial component of a divergent mRNA export pathway in *Toxoplasma gondii*. Mol. Microbiol. 102, 672–689 10.1111/mmi.13485 27542978 PMC5118106

[114] Serpeloni, M., Vidal, N.M., Goldenberg, S., Avila, A.R. and Hoffmann, F.G. (2011) Comparative genomics of proteins involved in RNA nucleocytoplasmic export. BMC Evol. Biol. 11, 7 10.1186/1471-2148-11-7 21223572 PMC3032688

[115] Rajakyla, E.K., Viita, T., Kyheroinen, S., Huet, G., Treisman, R. and Vartiainen, M.K. (2015) RNA export factor Ddx19 is required for nuclear import of the SRF coactivator MKL1. Nat. Commun. 6, 5978 10.1038/ncomms6978 25585691 PMC4309436

[116] Kaminski, T., Siebrasse, J.P. and Kubitscheck, U. (2013) A single molecule view on Dbp5 and mRNA at the nuclear pore. Nucleus 4, 8–13 10.4161/nucl.23386 23324459 PMC3585031

[117] Folkmann, A.W., Noble, K.N., Cole, C.N. and Wente, S.R. (2011) Dbp5, Gle1-IP6 and Nup159: a working model for mRNP export. Nucleus 2, 540–548 10.4161/nucl.2.6.17881 22064466 PMC3324343

[118] Arul Nambi Rajan, A. and Montpetit, B. (2021) Emerging molecular functions and novel roles for the DEAD-box protein Dbp5/DDX19 in gene expression. Cell. Mol. Life Sci. 78, 2019–2030 10.1007/s00018-020-03680-y 33205304 PMC7969391

[119] Lin, D.H., Correia, A.R., Cai, S.W., Huber, F.M., Jette, C.A. and Hoelz, A. (2018) Structural and functional analysis of mRNA export regulation by the nuclear pore complex. Nat. Commun. 9, 2319 10.1038/s41467-018-04459-3 29899397 PMC5998080

[120] Kendirgi, F., Barry, D.M., Griffis, E.R., Powers, M.A. and Wente, S.R. (2003) An essential role for hGle1 nucleocytoplasmic shuttling in mRNA export. J. Cell Biol. 160, 1029–1040 10.1083/jcb.200211081 12668658 PMC2172758

[121] Braud, C., Zheng, W. and Xiao, W. (2013) Identification and analysis of LNO1-like and AtGLE1-like nucleoporins in plants. Plant Signal. Behav. 8, e27376 10.4161/psb.27376 24384931 PMC4091346

[122] Lee, H.S., Lee, D.H., Cho, H.K., Kim, S.H., Auh, J.H. and Pai, H.S. (2015) InsP6-sensitive variants of the Gle1 mRNA export factor rescue growth and fertility defects of the ipk1 low-phytic-acid mutation in Arabidopsis. Plant Cell 27, 417–431 10.1105/tpc.114.132134 25670768 PMC4456929

[123] Imai, A., Ohtani, M., Nara, A., Tsukakoshi, A., Narita, A., Hirakawa, H. et al. (2020) The *Lotus japonicus* nucleoporin GLE1 is involved in symbiotic association with rhizobia. Physiol. Plant. 168, 590–600 10.1111/ppl.12996 31115057

[124] Lee, J.Y., Lee, H.S., Wi, S.J., Park, K.Y., Schmit, A.C. and Pai, H.S. (2009) Dual functions of *Nicotiana benthamiana* Rae1 in interphase and mitosis. Plant J. 59, 278–291 10.1111/j.1365-313X.2009.03869.x 19392703

[125] Irwin, N.A.T. and Keeling, P.J. (2019) Extensive reduction of the nuclear pore complex in nucleomorphs. Genome Biol. Evol. 11, 678–687 10.1093/gbe/evz029 30715330 PMC6411479

[126] Pazos, F. and Valencia, A. (2008) Protein co-evolution, co-adaptation and interactions. EMBO J. 27, 2648–2655 10.1038/emboj.2008.189 18818697 PMC2556093

[127] Gong, S., Worth, C.L., Bickerton, G.R., Lee, S., Tanramluk, D. and Blundell, T.L. (2009) Structural and functional restraints in the evolution of protein families and superfamilies. Biochem. Soc. Trans. 37, 727–733 10.1042/BST0370727 19614584

[128] Bapteste, E., Charlebois, R.L., MacLeod, D. and Brochier, C. (2005) The two tempos of nuclear pore complex evolution: highly adapting proteins in an ancient frozen structure. Genome Biol. 6, R85 10.1186/gb-2005-6-10-r85 16207356 PMC1257468

[129] Worth, C.L., Gong, S. and Blundell, T.L. (2009) Structural and functional constraints in the evolution of protein families. Nat. Rev. Mol. Cell Biol. 10, 709–720 10.1038/nrm2762 19756040

[130] Pal, C., Papp, B. and Lercher, M.J. (2006) An integrated view of protein evolution. Nat. Rev. Genet. 7, 337–348 10.1038/nrg1838 16619049

[131] D'Angelo, M.A., Gomez-Cavazos, J.S., Mei, A., Lackner, D.H. and Hetzer, M.W. (2012) A change in nuclear pore complex composition regulates cell differentiation. Dev. Cell 22, 446–458 10.1016/j.devcel.2011.11.021 22264802 PMC3288503

[132] Rosenblum, J.S. and Blobel, G. (1999) Autoproteolysis in nucleoporin biogenesis. Proc. Natl Acad. Sci. U.S.A. 96, 11370–11375 10.1073/pnas.96.20.11370 10500183 PMC18040

[133] Griffis, E.R., Xu, S. and Powers, M.A. (2003) Nup98 localizes to both nuclear and cytoplasmic sides of the nuclear pore and binds to two distinct nucleoporin subcomplexes. Mol. Biol. Cell 14, 600–610 10.1091/mbc.e02-09-0582 12589057 PMC149995

[134] Teixeira, M.T., Fabre, E. and Dujon, B. (1999) Self-catalyzed cleavage of the yeast nucleoporin Nup145p precursor. J. Biol. Chem. 274, 32439–32444 10.1074/jbc.274.45.32439 10542288

[135] Allegretti, M., Zimmerli, C.E., Rantos, V., Wilfling, F., Ronchi, P., Fung, H.K.H. et al. (2020) In-cell architecture of the nuclear pore and snapshots of its turnover. Nature 586, 796–800 10.1038/s41586-020-2670-5 32879490

[136] Mahamid, J., Pfeffer, S., Schaffer, M., Villa, E., Danev, R., Cuellar, L.K. et al. (2016) Visualizing the molecular sociology at the HeLa cell nuclear periphery. Science 351, 969–972 10.1126/science.aad8857 26917770

[137] Liashkovich, I., Meyring, A., Kramer, A. and Shahin, V. (2011) Exceptional structural and mechanical flexibility of the nuclear pore complex. J. Cell Physiol. 226, 675–682 10.1002/jcp.22382 20717933

[138] Jaggi, R.D., Franco-Obregon, A., Muhlhausser, P., Thomas, F., Kutay, U. and Ensslin, K. (2003) Modulation of nuclear pore topology by transport modifiers. Biophys. J. 84, 665–670 10.1016/S0006-3495(03)74886-3 12524319 PMC1302647

[139] Koh, J. and Blobel, G. (2015) Allosteric regulation in gating the central channel of the nuclear pore complex. Cell 161, 1361–1373 10.1016/j.cell.2015.05.013 26046439

[140] Blus, B.J., Koh, J., Krolak, A., Seo, H.S., Coutavas, E. and Blobel, G. (2019) Allosteric modulation of nucleoporin assemblies by intrinsically disordered regions. Sci. Adv. 5, eaax1836 10.1126/sciadv.aax1836 31807700 PMC6881172

[141] Feldherr, C.M. and Akin, D. (1990) The permeability of the nuclear envelope in dividing and nondividing cell cultures. J. Cell Biol. 111, 1–8 10.1083/jcb.111.1.1 PMC2116163 2365731

[142] Elosegui-Artola, A., Andreu, I., Beedle, A.E.M., Lezamiz, A., Uroz, M., Kosmalska, A.J. et al. (2017) Force triggers YAP nuclear entry by regulating transport across nuclear pores. Cell 171, 1397–1410.e14 10.1016/j.cell.2017.10.008 29107331

[143] Guttinger, S., Laurell, E. and Kutay, U. (2009) Orchestrating nuclear envelope disassembly and reassembly during mitosis. Nat. Rev. Mol. Cell Biol. 10, 178–191 10.1038/nrm2641 19234477

[144] Pradillo, M., Evans, D. and Graumann, K. (2019) The nuclear envelope in higher plant mitosis and meiosis. Nucleus 10, 55–66 10.1080/19491034.2019.1587277 30879391 PMC6527396

[145] Cross, F.R. and Umen, J.G. (2015) The Chlamydomonas cell cycle. Plant J. 82, 370–392 10.1111/tpj.12795 25690512 PMC4409525

[146] Ali, E.I., Loidl, J. and Howard-Till, R.A. (2018) A streamlined cohesin apparatus is sufficient for mitosis and meiosis in the protist Tetrahymena. Chromosoma 127, 421–435 10.1007/s00412-018-0673-x 29948142 PMC6208729

[147] Ogbadoyi, E., Ersfeld, K., Robinson, D., Sherwin, T. and Gull, K. (2000) Architecture of the *Trypanosoma brucei* nucleus during interphase and mitosis. Chromosoma 108, 501–513 10.1007/s004120050402 10794572

[148] Champion, L., Linder, M.I. and Kutay, U. (2017) Cellular reorganization during mitotic entry. Trends Cell Biol. 27, 26–41 10.1016/j.tcb.2016.07.004 27528558

[149] Dultz, E., Zanin, E., Wurzenberger, C., Braun, M., Rabut, G., Sironi, L. et al. (2008) Systematic kinetic analysis of mitotic dis- and reassembly of the nuclear pore in living cells. J. Cell Biol. 180, 857–865 10.1083/jcb.200707026 18316408 PMC2265396

[150] Dey, G., Culley, S., Curran, S., Schmidt, U., Henriques, R., Kukulski, W. et al. (2020) Closed mitosis requires local disassembly of the nuclear envelope. Nature 585, 119–123 10.1038/s41586-020-2648-3 32848252 PMC7610560

[151] Exposito-Serrano, M., Sanchez-Molina, A., Gallardo, P., Salas-Pino, S. and Daga, R.R. (2020) Selective nuclear pore complex removal drives nuclear envelope division in fission yeast. Curr. Biol. 30, 3212–3222.e2 10.1016/j.cub.2020.05.066 32502403

[152] Kaiser, C.A. and Schekman, R. (1990) Distinct sets of SEC genes govern transport vesicle formation and fusion early in the secretory pathway. Cell 61, 723–733 10.1016/0092-8674(90)90483-U 2188733

[153] Dokudovskaya, S., Waharte, F., Schlessinger, A., Pieper, U., Devos, D.P., Cristea, I.M. et al. (2011) A conserved coatomer-related complex containing Sec13 and Seh1 dynamically associates with the vacuole in *Saccharomyces cerevisiae*. Mol. Cell. Proteom. 10, M110 006478 10.1074/mcp.M110.006478 PMC3108837 21454883

[154] Itoh, G., Sugino, S., Ikeda, M., Mizuguchi, M., Kanno, S., Amin, M.A. et al. (2013) Nucleoporin Nup188 is required for chromosome alignment in mitosis. Cancer Sci. 104, 871–879 10.1111/cas.12159 23551833 PMC7657133

[155] Hashizume, C., Moyori, A., Kobayashi, A., Yamakoshi, N., Endo, A. and Wong, R.W. (2013) Nucleoporin Nup62 maintains centrosome homeostasis. Cell Cycle 12, 3804–3816 10.4161/cc.26671 24107630 PMC3905072

[156] Zuccolo, M., Alves, A., Galy, V., Bolhy, S., Formstecher, E., Racine, V. et al. (2007) The human Nup107-160 nuclear pore subcomplex contributes to proper kinetochore functions. EMBO J. 26, 1853–1864 10.1038/sj.emboj.7601642 17363900 PMC1847668

[157] Belgareh, N., Rabut, G., Bai, S.W., van Overbeek, M., Beaudouin, J., Daigle, N. et al. (2001) An evolutionarily conserved NPC subcomplex, which redistributes in part to kinetochores in mammalian cells. J. Cell Biol. 154, 1147–1160 10.1083/jcb.200101081 11564755 PMC2150808

[158] Loiodice, I., Alves, A., Rabut, G., Van Overbeek, M., Ellenberg, J., Sibarita, J.B. et al. (2004) The entire Nup107-160 complex, including three new members, is targeted as one entity to kinetochores in mitosis. Mol. Biol. Cell 15, 3333–3344 10.1091/mbc.e03-12-0878 15146057 PMC452587

[159] Platani, M., Santarella-Mellwig, R., Posch, M., Walczak, R., Swedlow, J.R. and Mattaj, I.W. (2009) The Nup107-160 nucleoporin complex promotes mitotic events via control of the localization state of the chromosome passenger complex. Mol. Biol. Cell 20, 5260–5275 10.1091/mbc.e09-05-0377 19864462 PMC2793300

[160] Platani, M., Samejima, I., Samejima, K., Kanemaki, M.T. and Earnshaw, W.C. (2018) Seh1 targets GATOR2 and Nup153 to mitotic chromosomes. J. Cell Sci. 131, jcs213140 10.1242/jcs.213140 29618633 PMC5992584

[161] Davis, L.I. and Blobel, G. (1987) Nuclear pore complex contains a family of glycoproteins that includes p62: glycosylation through a previously unidentified cellular pathway. Proc. Natl Acad. Sci. U.S.A. 84, 7552–7556 10.1073/pnas.84.21.7552 3313397 PMC299337

[162] Kuhn, T.M., Pascual-Garcia, P., Gozalo, A., Little, S.C. and Capelson, M. (2019) Chromatin targeting of nuclear pore proteins induces chromatin decondensation. J. Cell Biol. 218, 2945–2961 10.1083/jcb.201807139 31366666 PMC6719443

[163] Capelson, M., Liang, Y., Schulte, R., Mair, W., Wagner, U. and Hetzer, M.W. (2010) Chromatin-bound nuclear pore components regulate gene expression in higher eukaryotes. Cell 140, 372–383 10.1016/j.cell.2009.12.054 20144761 PMC2821818

[164] Kalverda, B., Pickersgill, H., Shloma, V.V. and Fornerod, M. (2010) Nucleoporins directly stimulate expression of developmental and cell-cycle genes inside the nucleoplasm. Cell 140, 360–371 10.1016/j.cell.2010.01.011 20144760

[165] Vaquerizas, J.M., Suyama, R., Kind, J., Miura, K., Luscombe, N.M. and Akhtar, A. (2010) Nuclear pore proteins nup153 and megator define transcriptionally active regions in the Drosophila genome. PLoS Genet. 6, e1000846 10.1371/journal.pgen.1000846 20174442 PMC2820533

[166] Franks, T.M., McCloskey, A., Shokirev, M.N., Benner, C., Rathore, A. and Hetzer, M.W. (2017) Nup98 recruits the Wdr82-Set1A/COMPASS complex to promoters to regulate H3K4 trimethylation in hematopoietic progenitor cells. Genes Dev. 31, 2222–2234 10.1101/gad.306753.117 29269482 PMC5769767

[167] Light, W.H., Freaney, J., Sood, V., Thompson, A., D'Urso, A., Horvath, C.M. et al. (2013) A conserved role for human Nup98 in altering chromatin structure and promoting epigenetic transcriptional memory. PLoS Biol. 11, e1001524 10.1371/journal.pbio.1001524 23555195 PMC3608542

[168] Ibarra, A., Benner, C., Tyagi, S., Cool, J. and Hetzer, M.W. (2016) Nucleoporin-mediated regulation of cell identity genes. Genes Dev. 30, 2253–2258 10.1101/gad.287417.116 27807035 PMC5110992

[169] Iglesias, N., Paulo, J.A., Tatarakis, A., Wang, X., Edwards, A.L., Bhanu, N.V. et al. (2020) Native chromatin proteomics reveals a role for specific nucleoporins in heterochromatin organization and maintenance. Mol. Cell 77, 51–66.e8 10.1016/j.molcel.2019.10.018 31784357 PMC7224636

[170] Gozalo, A., Duke, A., Lan, Y., Pascual-Garcia, P., Talamas, J.A., Nguyen, S.C. et al. (2020) Core components of the nuclear pore bind distinct states of chromatin and contribute to polycomb repression. Mol. Cell 77, 67–81.e7 10.1016/j.molcel.2019.10.017 31784359 PMC7439457

[171] Van de Vosse, D.W., Wan, Y., Lapetina, D.L., Chen, W.M., Chiang, J.H., Aitchison, J.D. et al. (2013) A role for the nucleoporin Nup170p in chromatin structure and gene silencing. Cell 152, 969–983 10.1016/j.cell.2013.01.049 23452847 PMC3690833

[172] Kehat, I., Accornero, F., Aronow, B.J. and Molkentin, J.D. (2011) Modulation of chromatin position and gene expression by HDAC4 interaction with nucleoporins. J. Cell Biol. 193, 21–29 10.1083/jcb.201101046 21464227 PMC3082185

[173] Smith, S., Galinha, C., Desset, S., Tolmie, F., Evans, D., Tatout, C. et al. (2015) Marker gene tethering by nucleoporins affects gene expression in plants. Nucleus 6, 471–478 10.1080/19491034.2015.1126028 26652762 PMC4915490

[174] Kee, H.L., Dishinger, J.F., Blasius, T.L., Liu, C.J., Margolis, B. and Verhey, K.J. (2012) A size-exclusion permeability barrier and nucleoporins characterize a ciliary pore complex that regulates transport into cilia. Nat. Cell Biol. 14, 431–437 10.1038/ncb2450 22388888 PMC3319646

[175] Endicott, S.J. and Brueckner, M. (2018) NUP98 sets the size-exclusion diffusion limit through the ciliary base. Curr. Biol. 28, 1643–1650.e3 10.1016/j.cub.2018.04.014 29731308 PMC7106777

[176] Del Viso, F., Huang, F., Myers, J., Chalfant, M., Zhang, Y., Reza, N. et al. (2016) Congenital heart disease genetics uncovers context-dependent organization and function of nucleoporins at cilia. Dev. Cell 38, 478–492 10.1016/j.devcel.2016.08.002 27593162 PMC5021619

[177] Marquez, J., Bhattacharya, D., Lusk, C.P. and Khokha, M.K. (2021) Nucleoporin NUP205 plays a critical role in cilia and congenital disease. Dev. Biol. 469, 46–53 10.1016/j.ydbio.2020.10.001 33065118 PMC7722132

[178] Nachury, M.V., Seeley, E.S. and Jin, H. (2010) Trafficking to the ciliary membrane: how to get across the periciliary diffusion barrier? Annu. Rev. Cell Dev. Biol. 26, 59–87 10.1146/annurev.cellbio.042308.113337 19575670 PMC2952038

[179] Dishinger, J.F., Kee, H.L., Jenkins, P.M., Fan, S., Hurd, T.W., Hammond, J.W. et al. (2010) Ciliary entry of the kinesin-2 motor KIF17 is regulated by importin-beta2 and RanGTP. Nat. Cell Biol. 12, 703–710 10.1038/ncb2073 20526328 PMC2896429

[180] Hurd, T.W., Fan, S. and Margolis, B.L. (2011) Localization of retinitis pigmentosa 2 to cilia is regulated by Importin beta2. J. Cell Sci. 124(Pt 5), 718–726 10.1242/jcs.070839 21285245 PMC3039017

[181] Breslow, D.K., Koslover, E.F., Seydel, F., Spakowitz, A.J. and Nachury, M.V. (2013) An in vitro assay for entry into cilia reveals unique properties of the soluble diffusion barrier. J. Cell Biol. 203, 129–147 10.1083/jcb.201212024 24100294 PMC3798247

[182] Salisbury, J.L., Suino, K.M., Busby, R. and Springett, M. (2002) Centrin-2 is required for centriole duplication in mammalian cells. Curr. Biol. 12, 1287–1292 10.1016/S0960-9822(02)01019-9 12176356

[183] Resendes, K.K., Rasala, B.A. and Forbes, D.J. (2008) Centrin 2 localizes to the vertebrate nuclear pore and plays a role in mRNA and protein export. Mol. Cell. Biol. 28, 1755–1769 10.1128/MCB.01697-07 18172010 PMC2258798

[184] Ludwig, M. and Gibbs, S.P. (1989) Evidence that the nucleomorphs of chlorarachnion reptans (chlorarachniophyceae) are vestigial nuclei: morphology, division and DNA-DAPI fluorescence. J. Phycol. 25, 385–394 10.1111/j.1529-8817.1989.tb00135.x

[185] Jovanovic-Talisman, T., Tetenbaum-Novatt, J., McKenney, A.S., Zilman, A., Peters, R., Rout, M.P. et al. (2009) Artificial nanopores that mimic the transport selectivity of the nuclear pore complex. Nature 457, 1023–1027 10.1038/nature07600 19098896 PMC2764719

[186] Fragasso, A., de Vries, H.W., Andersson, J., van der Sluis, E.O., van der Giessen, E., Dahlin, A. et al. (2021) A designer FG-Nup that reconstitutes the selective transport barrier of the nuclear pore complex. Nat. Commun. 12, 2010 10.1038/s41467-021-22293-y 33790297 PMC8012357

[187] Panatala, R., Barbato, S., Kozai, T., Luo, J., Kapinos, L.E. and Lim, R.Y.H. (2019) Nuclear pore membrane proteins self-assemble into nanopores. Biochemistry 58, 484–488 10.1021/acs.biochem.8b01179 30605322

